# National cost of intensive care in Japan from 2018 to 2022

**DOI:** 10.1186/s40560-026-00868-5

**Published:** 2026-03-04

**Authors:** Hiroyuki Ohbe, Daisuke Kudo, Shigeki Kushimoto, Yudai Iwasaki, Takuya Shiga, Rei Goto, Kei Nishiyama, Ryutarou Seo, Masao Iwagami, Toshikazu Abe, Yuya Kimura, Hiroki Matsui, Kiyohide Fushimi, Hideo Yasunaga

**Affiliations:** 1 Department of Emergency and Critical Care Medicine, Tohoku University Hospital, 1-1 Seiryo-machi, Aoba-ku, Sendai, 980-8574 Japan; 2 Department of Clinical Epidemiology and Health Economics, School of Public Health, The University of Tokyo, 7-3-1 Hongo, Bunkyo-ku, Tokyo, 113-0033 Japan; 3 Division of Emergency and Critical Care Medicine, Tohoku University Graduate School of Medicine, 2-1 Seiryo-machi, Aoba-ku, Sendai, Miyagi 980-8575 Japan; 4 Kawasaki Saiwai Hospital, 31-27 Omiya-cho, Saiwai-ku, Kawasaki, Kanagawa 212-0014 Japan; 5 Department of Anesthesiology and Perioperative Medicine, Tohoku University Graduate School of Medicine, 2-1 Seiryo-machi, Aoba-ku, Sendai, Miyagi 980-8575 Japan; 6 Experience Design and Alliance Section, Tohoku University Hospital, Sendai, Miyagi Japan; 7 Graduate School of Business Administration, Keio University, Yokohama, Kanagawa Japan; 8 Department of Emergency and Critical Care, Niigata University, 1-754 Asahimachidori, Chuo-ku, Niigata, 951-8510 Japan; 9 Intensive Care Unit, Kobe City Medical Center General Hospital, Hyogo, Japan; 10 Department of Digital Health, Social Medicine Group, Institute of Medicine, University of Tsukuba, Tsukuba, Japan; 11 Department of Emergency and Critical Care Medicine, Tsukuba Memorial Hospital, Tsukuba, Japan; 12 Department of Health Services Research, Graduate School of Medicine, The University of Tokyo, 7-3-1 Hongo, Bunkyo-ku, Tokyo, 113-0033 Japan; 13 Department of Health Policy and Informatics, Institute of Science Tokyo Graduate School, 2-12-1 Ookayama, Meguro-ku, Tokyo, 152-8550 Japan

**Keywords:** Intensive care unit, Critical care costs, Health expenditure, Macroeconomic impact, Japan

## Abstract

**Background:**

Intensive care is a cornerstone of modern health systems, yet it remains among the most resource-intensive and costly forms of care. While intensive care unit (ICU) costs represent a substantial portion of health care expenditures in many high-income countries, national-level data on ICU costs in Japan have been lacking. We aimed to estimate national ICU costs in Japan using administrative and hospital-level data, employing internationally comparable methods.

**Methods:**

We conducted a nationwide retrospective cohort study using the Diagnosis Procedure Combination Study Group database and the Hospital Bed Function Report during fiscal years 2018–2022. ICU costs were estimated using a bottom-up costing approach aligned with the method used in major international studies, and focused on certified adult and pediatric ICUs. Macroeconomic impact, temporal trends, and regional variation were assessed. We also analyzed the cost of intermediate care units (IMCU) and IMCU–ICU ratio.

**Results:**

A total of 1,453,929 ICU patients were identified in the DPC Study Group database from 2018 to 2022, which covers 68.2% of all ICU beds in Japan. The mean ICU cost per patient-day was ¥197,277 (approximately $1,793 USD), and the estimated national ICU cost over the 2018–2022 period was ¥1,785 billion (mean ¥357 billion per year). This represented 0.065% of nominal gross domestic product (GDP), 0.56% of total health expenditures, 1.42% of hospital expenditures, and 2.17% of inpatient expenditures. The macroeconomic impact of ICU care was stable from 2018 to 2022. The proportion of ICU costs relative to nominal GDP, total health expenditures, and hospital expenditures in Japan was substantially lower than in the United States, Canada, and Australia. Regional variation was pronounced, with up to a 7.5-fold difference across prefectures. The estimated national IMCU cost over the 2018–2022 period was ¥2,838 billion (mean ¥568 billion per year), approximately 1.5 times greater than ICU cost (IMCU–ICU ratio is approximately 1.59).

**Conclusions:**

This study provides the first comprehensive national estimates of ICU costs in Japan using internationally aligned methods. These findings indicate lower ICU spending in Japan than in other high-income countries.

**Supplementary Information:**

The online version contains supplementary material available at 10.1186/s40560-026-00868-5.

## Background

Intensive care is a cornerstone of modern health care, providing life-sustaining support for critically ill patients and requiring complex monitoring, organ support, and multidisciplinary management. However, care in the intensive care unit (ICU) is among the most resource-intensive and expensive aspects of hospital medicine, accounting for a considerable proportion of health care expenditures in high-income countries [1, 2].

Previous studies have reported that ICU care accounts for approximately 8.1–20% of hospital expenditures, 1.4–11% of total health expenditures, and 0.15–0.8% of gross domestic product (GDP) in the United States, Canada, Belgium, and Australia [1–6]. These figures underscore the substantial economic impact of ICU. Understanding ICU costs—including their scale, trends, and proportion of national health spending—is crucial for health policy, resource allocation, and evaluating system sustainability and efficiency, as well as for facilitating international comparisons.

Compared with the United States and Europe, evidence on ICU costs in Japan remains limited. Although several studies have examined ICU costs in Japan [7–9], these were either single-center financial analyses [7] or cost-effectiveness analyses of intensivist staffing and intermediate care units (IMCU) [8, 9]. None have provided national ICU cost estimates. Given Japan’s rapidly aging population and rising health care demands, ICU demand is expected to increase, because older adults require more critical care [10, 11]. Robust national-level data on ICU costs—benchmarked against international standards—are essential for resource allocation, policy development, and global health system comparisons.

This study aimed to provide the first nationwide estimation of intensive care costs in Japan using administrative claims and hospital-level data, with definitions and costing approaches consistent with those used in major international studies.

## Methods

### Study design and data source

We conducted a nationwide, retrospective cohort study using two data sources: the Diagnosis Procedure Combination (DPC) Study Group database and the Hospital Bed Function Report from fiscal years 2018–2022 [12, 13]. The DPC Study Group database includes discharge abstract data (Format 1), administrative claims data (EF files), and hospital-level data (Format 3) from more than 1500 voluntarily participating acute-care hospitals, representing over 50% of acute-care hospital beds nationwide [12]. A previous validation study of this database revealed that the sensitivity and specificity of EF file procedures exceeded 90% each [14]. We also used the Hospital Bed Function Report, published annually by the Ministry of Health, Labour, and Welfare of Japan, which provides functional and statistical information on hospitals as of July 1 each year [13].

This study was approved by the Institutional Review Board of the University of Tokyo (Approval Number: 3501–5; Approval Date: May 19, 2021). Because all data were de-identified, the requirement for informed consent was waived. This study adhered to the principles of the Declaration of Helsinki.

All methodological details are provided in Supplementary Methods.

### ICU cost estimation framework

**Fig. 1:**
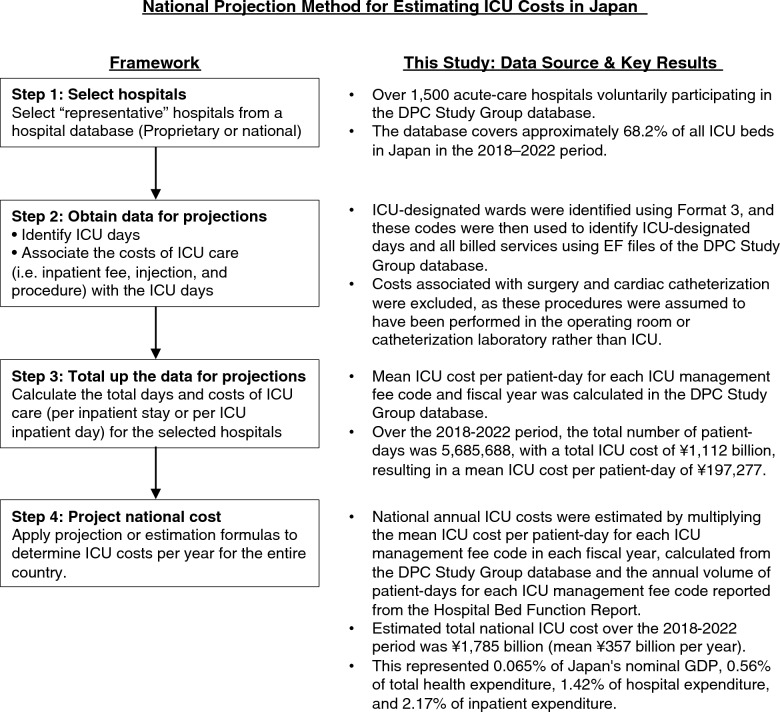
National projection method for estimating ICU costs in Japan. *ICU* Intensive care unit, *DPC* Diagnosis procedure combination, *GDP* Gross domestic product

To estimate the national ICU costs in Japan, we applied a bottom-up costing approach based on the national projection method described by Halpern and Pastores [15]. An overview of this approach is shown in Fig. 1.

The analytic perspective of this study was that of the payer, with the objective of informing policy evaluation and resource allocation. Accordingly, we used charged costs based on reference prices in Japan’s national fee schedule and derived from administrative claims data as our primary cost measure. Because Japan’s national fee schedule is periodically revised, the charged costs closely approximate the accounted costs incurred by hospitals [16].

### Definition of ICU cost and cost calculation

The primary outcome of this study was the national ICU cost, estimated using definitions and methods consistent with those in major previous ICU cost studies [1, 2, 17]. In this study, an ICU was defined as an adult or pediatric unit formally certified by the Japanese Society of Intensive Care Medicine [18]. Costs of IMCUs—also known as high-dependency units, high-care units, or step-down units—were excluded. Additional details and the procedure codes used to define the ICUs are provided in Supplementary Table 1. When calculating ICU costs, we excluded billed charges for surgery and cardiac catheterization, as these procedures were assumed to have been performed in the operating room or catheterization laboratory rather than the ICU.

The mean ICU cost per patient-day was calculated by dividing the total cost by the total number of patient-days for each ICU management fee code and fiscal year. National ICU costs were then estimated by multiplying the average cost per patient-day for each ICU management fee code and fiscal year by the annual volume of patient-days for that code, as reported in the Hospital Bed Function Report [13], yielding total annual national ICU costs.

### Macroeconomic impact, regional variation, and detailed cost breakdown

We evaluated national ICU costs as a proportion of key macroeconomic and health expenditure indicators for each year. These indicators included nominal GDP, OECD-defined total health expenditure, OECD-defined hospital expenditures, and national inpatient expenditure as defined by the Ministry of Health, Labour and Welfare of Japan. These proportions allow evaluation of the relative financial priority assigned to intensive care in the context of national economic capacity (GDP), overall health care spending (total health expenditure), and hospital resource allocation (hospital and inpatient expenditures). Data for these indicators were obtained from OECD Health Statistics [19] and official Japanese government sources [20]. When comparable national ICU cost data from other countries were available from published studies, we derived their nominal GDP, OECD-defined total health expenditure, and OECD-defined hospital expenditures from OECD Health Statistics [18] to enable cross-country comparison of relative financial priorities.

We also examined regional variation by estimating ICU costs per 100,000 population for each of Japan’s 47 prefectures during 2018–2022. Prefecture-specific ICU costs were visualized using a choropleth map to illustrate geographic differences. We then assessed the correlation between prefecture-specific ICU costs and ICU bed density (beds per 100,000 population) using linear regression analysis and Pearson’s correlation coefficients.

To examine the composition of ICU costs, we conducted two types of cost breakdowns for the period 2018–2022. First, ICU costs were disaggregated into 10 specific categories based on classification code (*kubun* codes) defined in Japan’s national fee schedule. Second, ICU costs were disaggregated into each ICU management fee code, and the percentage contribution of each fee code to total costs was calculated.

### Analysis of IMCU costs

We also estimated national IMCU costs. The definition of IMCU in Japan is similar to that of the ICU, except that IMCUs are not required to have intensivist staffing or an ICU nurse, and the nurse-to-patient ratio is 1:3, 1:4, or 1:5. Additional details are provided in Supplementary Table 1. The same costing and aggregation procedures described above were applied. We also calculated combined national estimates for ICU and IMCU costs, and evaluated their macroeconomic impact, regional variation, and detailed cost breakdown.

### Statistical analyses

Continuous variables were summarized as means with standard deviation (SD) or medians with interquartile ranges (IQRs), as appropriate. Categorical variables were expressed as frequencies and percentages. To assess temporal trends, we performed linear regression analyses treating fiscal year as a continuous independent variable, and reported the p for trend based on the regression coefficient. A two-sided *p* value < 0.05 was considered statistically significant. All analyses were performed using Stata/SE, Version 19.0 (StataCorp, College Station, TX, USA).

## Results

A total of 1,453,929 ICU patients from 470 hospitals were identified in the DPC Study Group database from 2018 to 2022, corresponding to 24,278 ICU beds (calculated as the sum of each hospital’s ICU beds per year) (Table 1). Over this period, the total number of patient-days was 5,685,688, with a total ICU cost of \1,122 billion, yielding a mean ICU cost per patient-day of ¥197,277. Details for each ICU management fee code are provided in Supplementary Table 2.

**Table 1:** Annual trends in ICU costs from 2018 to 2022

Variable	Fiscal year						P for
	2018–2022	2018	2019	2020	2021	2022	trend
*ICU costs*							
DPC study group database							
Number of hospitals, *n*	470	343	413	389	410	375	0.58
Number of ICU beds, *n*	24,278	4022	5003	4994	5419	4840	0.25
Number of patients, *n*	1,453,929	252,512	317,241	299,094	302,885	282,197	0.64
Number of patient days, day	5,685,688	1,001,880	1,263,293	1,169,358	1,200,698	1,050,459	0.94
Total ICU costs, billion yen	1122	179	235	234	251	222	0.29
Mean cost per patient, yen	771,463	710,475	741,329	781,221	829,869	786,884	0.079
Mean cost per patient day, nominal, yen	197,277	179,067	186,164	199,818	209,341	211,390	0.004
Mean cost per patient day, real, yen	196,101	177,333	184,362	198,968	208,450	211,390	0.003
Hospital bed function report							
Number of hospitals, *n*	680	625	618	601	614	593	0.076
Number of ICU beds, *n*	35,586	7204	7134	7015	7221	7012	0.43
Number of patients, *n*	2,310,993	472,504	477,245	478,214	447,508	435,522	0.074
Number of patient days, day	9,167,865	1,892,731	1,928,662	1,813,177	1,732,102	1,801,193	0.13
Covering rate of ICU beds in DPC database, %	68.2	55.8	70.1	71.2	75.0	69.0	0.21
National estimated ICU cost, nominal, billion yen	1785	339	353	357	356	380	0.031
National estimated ICU cost, real, billion yen	1775	336	349	356	354	380	0.028
Macroeconomic impact							
Nominal GDP, billion yen	2,767,722	556,630	557,911	539,648	553,068	560,464	0.93
OECD-defined total health expenditure, billion yen	318,835	59,781	61,203	61,766	67,020	69,065	0.011
OECD-defined hospital expenditure, billion yen	125,301	24,264	25,144	24,281	25,211	26,401	0.13
National inpatient expenditure, billion yen	82,127	16,171	16,521	15,965	16,485	16,986	0.24
ICU cost per GDP, %	0.065	0.061	0.063	0.066	0.064	0.068	0.051
ICU cost per OECD-defined total health expenditure, %	0.56	0.57	0.58	0.58	0.53	0.55	0.23
ICU cost per OECD-defined hospital expenditure, %	1.42	1.40	1.40	1.47	1.41	1.44	0.43
ICU cost per national inpatient expenditure, %	2.17	2.09	2.14	2.24	2.16	2.24	0.13

*ICU* Intensive care unit, *DPC* Diagnosis Procedure Combination, *GDP* gross domestic product, *OECD* Organisation for Economic Co-operation and Development

According to the Hospital Bed Function Report from 2018 to 2022, there were 680 hospitals with a combined 35,586 ICU beds (summed annually across hospitals) meeting Japan’s ICU definition, with a total number of 9,167,865 ICU patient-days during the 5-year period (Table 1). Regarding the population representativeness, the DPC Study Group database covered 68.2% of all ICU beds nationwide over this period, using the Hospital Bed Function Report as the reference. Details for each ICU management fee code are presented in Supplementary Table 3. Characteristics of hospitals participating in and not participating in the DPC Study Group database among all hospitals with ICUs in Japan in 2022 are presented in Supplemental Table 4. Hospitals not participating in the DPC reimbursement system (i.e., those operating under a fee-for-service payment system) accounted for only 2.4% of all ICU beds nationwide; this proportion refers to reimbursement-system participation and is distinct from participation in the DPC Study Group database.

Applying the national projection method, the estimated total national ICU cost was ¥339 billion in 2018 and ¥380 billion in 2022 (Table 1 and Fig. 1). Over the 2018–2022 period, the cumulative estimated national ICU cost was ¥1,785 billion, corresponding to a mean of ¥357 billion per year. This represented 0.065% of nominal GDP, 0.56% of OECD-defined total health expenditure, 1.42% of OECD-defined hospital expenditures, and 2.17% of national inpatient expenditure. The mean ICU cost per patient-day, national estimated ICU cost, and OECD-defined total health expenditure showed significant upward trends over the study period (*p* for trend = 0.004, 0.031, and 0.011, respectively), whereas no significant trends were observed for the other variables (all *p* for trend > 0.05). Real costs closely paralleled the nominal costs, and the p-for-trend results were nearly identical.

Among the 470 hospitals with ICUs in the DPC Study Group database, the hospital-level proportion of ICU costs relative to total inpatient costs had a median of 4.3% (IQR, 3.2–6.1%).

Table 2 presents an international comparison of national ICU costs and related macroeconomic indicators across Japan, the United States [2], Canada [3], and Australia [6], based on national ICU cost data from previous studies. The proportion of ICU costs to nominal GDP, OECD-defined total health expenditure, and OECD-defined hospital expenditures in Japan was substantially lower than in these comparator countries.

**Table 2:** International comparison of national ICU costs and related macroeconomic indicators across Japan, the United States, Canada, and Australia

Variable	Japan	USA	Canada	Australian
Year	2022	2010	1986	2014
Cost indicators				
National currency	billion yen	billion USD	million C$	million A$
National estimated ICU cost	380	108	1030	2,119
Nominal GDP	560,464	14,964	505,670	1,661,739
OECD-defined total health expenditure	69,065	2604	41,066	159,628
OECD-defined hospital expenditure	26,401	815	17,637	64,492
Macroeconomic indicators				
OECD-defined total health expenditure per GDP, %	12.3%	17.4%	8.1%	9.6%
OECD-defined hospital expenditure per OECD-defined total health expenditure, %	38.2%	31.3%	42.9%	40.4%
ICU cost per GDP, %	0.065%	0.72%	0.20%	0.13%
ICU cost per OECD-defined total health expenditure, %	0.56%	4.1%	2.5%	1.3%
ICU cost per OECD-defined hospital expenditure, %	1.42%	13.3%	5.8%	3.3%

Cost indicators are presented in each country's original currency and are derived from published data; definitions and estimation methods may, therefore, vary across countries. Direct monetary comparisons of cost indicators were not the objective of this table. Instead, we contextualized published ICU cost estimates using each country’s GDP, OECD-defined total health expenditure, and OECD-defined hospital expenditures to illustrate how ICU spending fits within the macroeconomic context of each country

Nominal GDP, OECD-defined total health expenditure, and OECD-defined hospital expenditure are derived from OECD Health Statistics

*ICU* intensive care unit, *GDP* gross domestic product, *OECD* Organisation for Economic Co-operation and Development, *C$* Canadian dollar, *A$* Australian dollar

**Fig. 2:**
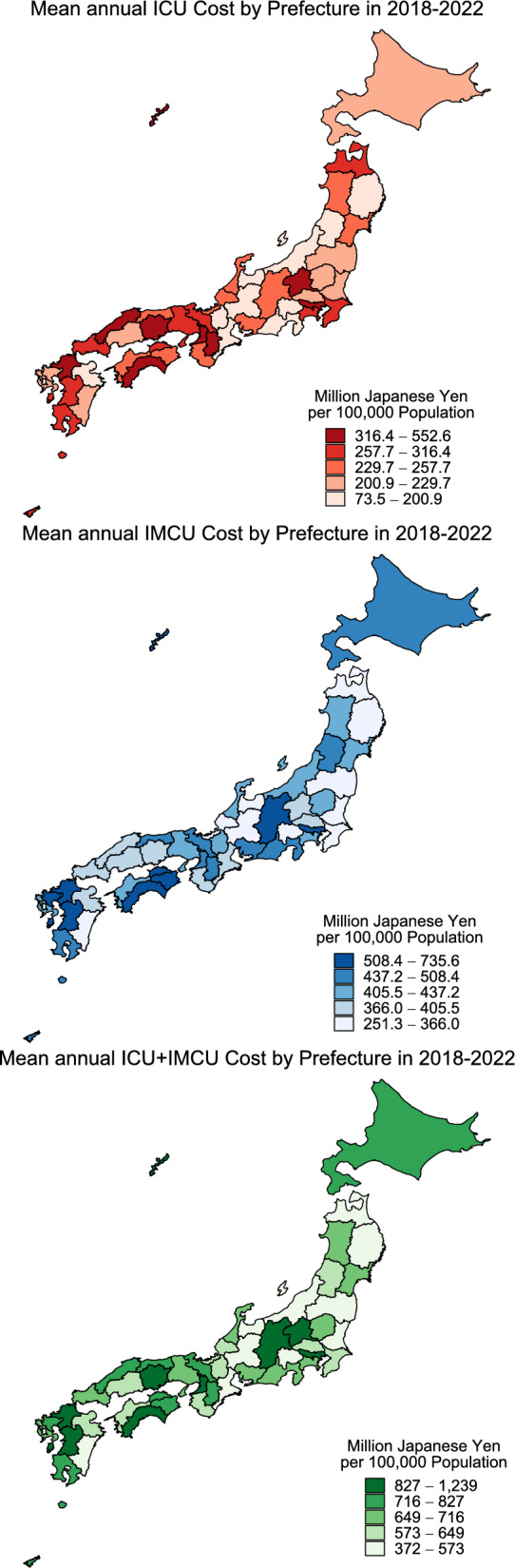
Regional variation in estimated ICU, IMCU, and ICU + IMCU costs per 100,000 population in Japan’s 47 prefectures, 2018–2022. *ICU* intensive care unit, *IMCU* intermediate care unit

ICU costs per 100,000 population varied significantly across Japan’s 47 prefectures, ranging from ¥73.5 to ¥552.6 million, representing 7.5-fold difference (maximum-to-minimum ratio) during 2018–2022 (Fig. 2 and Supplementary Table 5). Prefecture-specific ICU costs were strongly correlated with ICU bed density (*r* = 0.88, *p* < 0.001) (Supplemental Fig. 1).

**Fig. 3:**
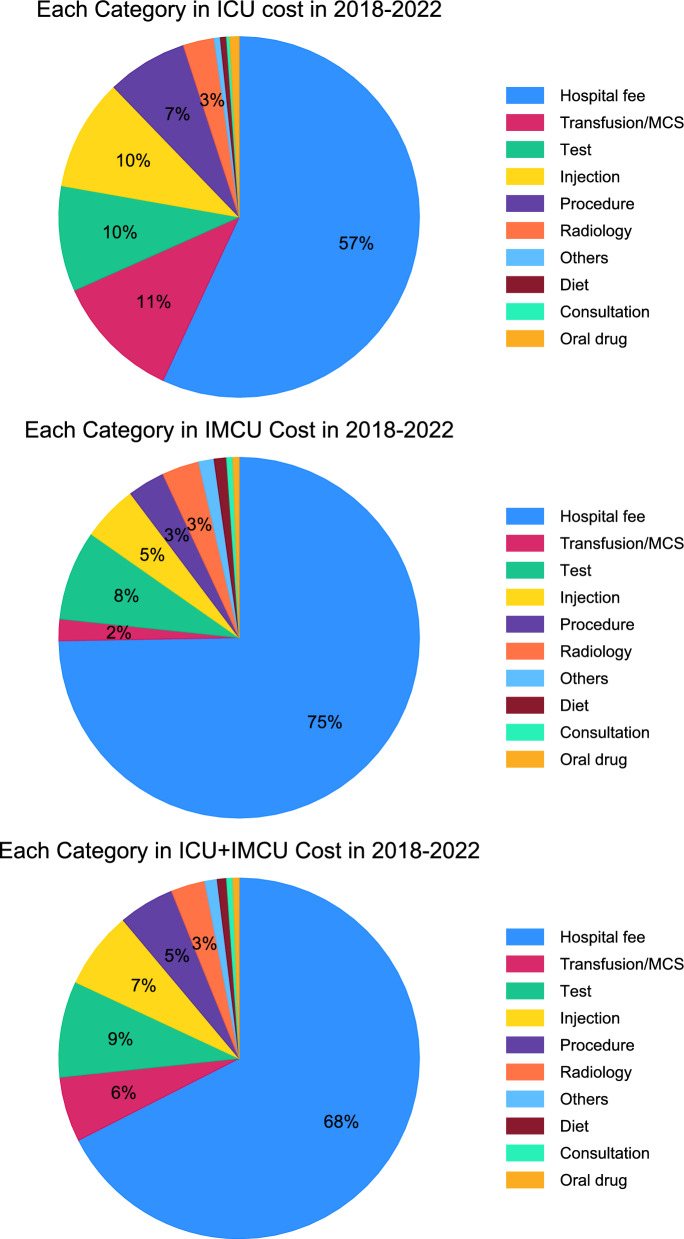
Composition of ICU, IMCU, and ICU + IMCU costs by ten specific cost categories, 2018–2022. Pie chart illustrating the composition of ICU, IMCU, and ICU + IMCU costs by ten specific cost categories: inpatient fee, transfusion/MCS, tests, injections, procedures, radiology, consultation, oral drugs, diet, and others, defined according to Japanese classification codes. *ICU* intensive care unit, *IMCU* intermediate care unit, *MCS* mechanical circulatory support

When broken down into ten specific cost categories, inpatient hospital fees accounted for the largest share (57%), followed by transfusion and mechanical circulatory support (11%), tests (10%), injections (10%), procedures (7%), and radiology (3%) (Fig. 3). The remaining categories—including other services, diet, consultation, and oral drugs—each contributed less than 1% of total costs.

**Fig. 4:**
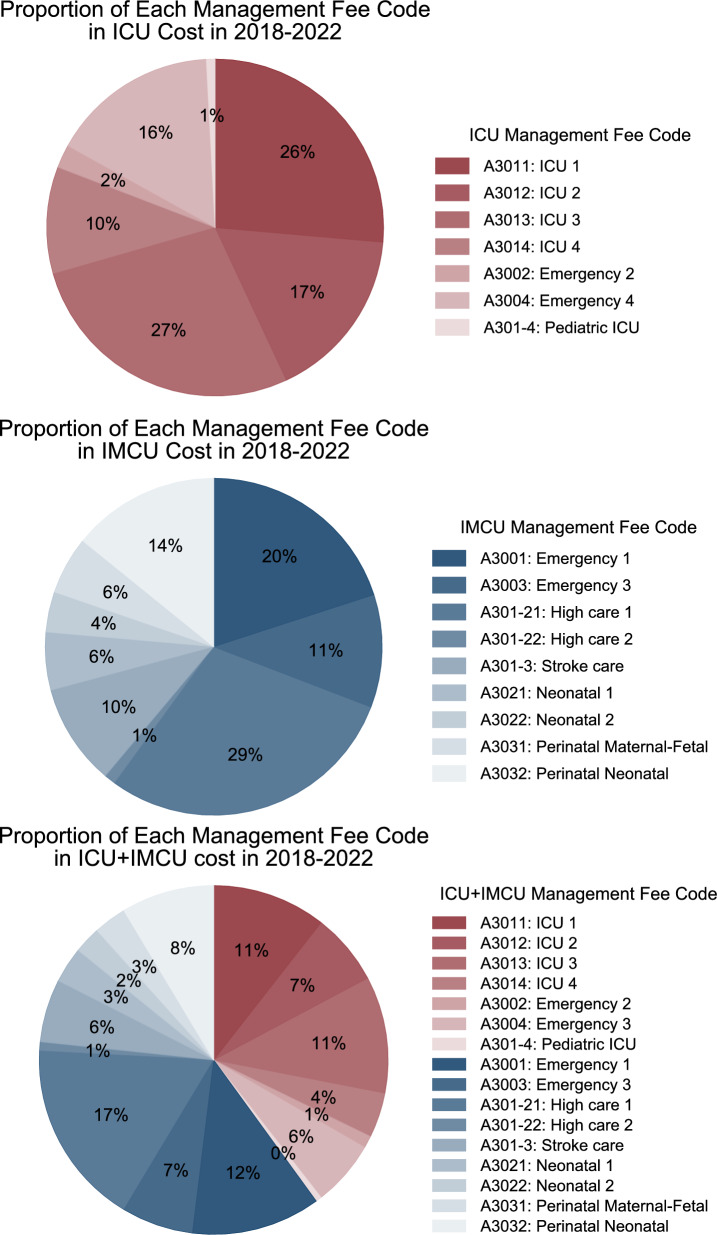
Breakdown of ICU, IMCU, and ICU + IMCU costs by management fee code, 2018–2022. *ICU* intensive care unit, *IMCU* intermediate care unit

A detailed breakdown of ICU management fee codes showed that the largest share of costs was attributed to ICU management fee 1–4 (81%) (Fig. 4). In contrast, emergency and critical care unit management fees 2 and 4 accounted for 18% and the pediatric ICU management fee contributed only 1%.

In the IMCU analysis, a total of 2,405,176 IMCU patients were identified (Table 3). The mean IMCU cost per patient-day during 2018 to 2022 was ¥119,342. The estimated total national IMCU cost was ¥502 billion in 2018 and ¥582 billion in 2022. Over the 2018–2022 period, the cumulative estimated national IMCU cost was ¥2,838 billion (mean ¥568 billion per year), compared with ¥1,785 billion for ICUs, yielding an IMCU–ICU cost ratio of approximately 1.59. Combined cumulative estimated national ICU and IMCU costs over the 2018–2022 period represented 0.17% of nominal GDP, 1.45% of OECD-defined total health expenditure, 3.69% of OECD-defined hospital expenditures, and 5.63% of national inpatient expenditure.

**Table 3:** Annual trends in IMCU and ICU + IMCU costs from 2018 to 2022

Variable	Fiscal year						*P* for
	2018–2022	2018	2019	2020	2021	2022	trend
*IMCU costs*							
DPC Study Group database							
Number of hospitals, *n*	668	465	527	543	563	509	0.36
Number of IMCU beds, *n*	56,361	9586	11,343	11,766	12,418	11,248	0.22
Number of patients, *n*	2,405,176	418,793	507,963	493,443	510,515	474,462	0.42
Number of patient days, day	14,143,631	2,517,419	3,012,110	2,954,222	3,019,188	2,640,692	0.78
Total IMCU costs, billion yen	1688	277	342	355	376	338	0.22
Mean cost per patient, yen	701,790	661,492	672,407	719,666	736,666	712,702	0.089
Mean cost per patient day, nominal, yen	119,342	110,044	113,395	120,206	124,563	128,054	0.001
Mean cost per patient day, real, yen	118,806	108,968	112,289	119,692	124,028	129,054	0.001
Hospital bed function report							
Number of hospitals, *n*	1172	1033	1047	1042	1074	1065	0.066
Number of IMCU beds, *n*	87,395	16,163	16,746	17,148	18,993	18,345	0.039
Number of patients, *n*	4,597,037	900,980	939,321	926,829	908,879	921,028	0.87
Number of patient days, day	23,281,413	4,396,689	4,663,173	5,089,141	4,602,406	4,530,004	0.84
Covering rate of IMCU beds in DPC database, %	64.5	59.3	67.7	68.6	65.4	61.3	0.92
National estimated IMCU cost, nominal, billion yen	2838	502	542	630	583	582	0.23
National estimated IMCU cost, real, billion yen	2825	498	537	627	581	582	0.21
Macroeconomic impact							
Nominal GDP, billion yen	2,767,722	556,630	557,911	539,648	553,068	560,464	0.93
OECD-defined total health expenditure, billion yen	318,835	59,781	61,203	61,766	67,020	69,065	0.011
OECD-defined hospital expenditure, billion yen	125,301	24,264	25,144	24,281	25,211	26,401	0.12
National inpatient expenditure, billion yen	82,127	16,171	16,521	15,965	16,485	16,986	0.24
IMCU cost per GDP, %	0.10	0.09	0.10	0.12	0.11	0.10	0.49
IMCU cost per OECD-defined total health expenditure, %	0.89	0.84	0.89	1.02	0.87	0.84	0.95
IMCU cost per OECD-defined hospital expenditure, %	2.27	2.07	2.15	2.59	2.31	2.20	0.59
IMCU cost per national inpatient expenditure, %	3.46	3.10	3.28	3.95	3.54	3.42	0.45
ICU + IMCU costs							
National estimated ICU + IMCU cost, nominal, billion yen	4623	840	895	987	939	962	0.12
National estimated ICU + IMCU cost, real, billion yen	4598	833	885	983	935	962	0.10
ICU + IMCU cost per GDP, %	0.17	0.15	0.16	0.18	0.17	0.17	0.19
ICU + IMCU cost per OECD-defined total health expenditure, %	1.45	1.41	1.46	1.60	1.40	1.39	0.770
ICU + IMCU cost per OECD-defined hospital expenditure, %	3.69	3.46	3.56	4.07	3.73	3.64	0.553
ICU + IMCU cost per national inpatient expenditure, %	5.63	5.20	5.41	6.18	5.70	5.66	0.369

*ICU* Intensive Care Unit, *IMCU* Intermediate Care Unit, *DPC* diagnosis procedure combination, *GDP* gross domestic product, *OECD* Organisation for Economic Co-operation and Development

## Discussion

This study provides the first national estimate of intensive care costs in Japan using routinely collected nationwide inpatient and hospital data, with ICU definitions and costing methods aligned to those used in major international studies. This approach enables robust, internationally comparable assessment of the economic burden of intensive care in Japan and establishes a methodological foundation for future research in this field. We found that the mean ICU cost per patient-day in Japan was ¥197,277 (approximately $1793 at an exchange rate of ¥110 per USD, which was the average rate during fiscal years 2018–2022). The estimated national ICU cost over the 2018–2022 period was ¥1785 billion (mean ¥357 billion per year), accounting for 0.065% of nominal GDP, 0.56% of OECD-defined total health expenditure, 1.42% of OECD-defined hospital expenditures, and 2.17% of national inpatient expenditures. Significant regional variation was observed, with ICU costs per 100,000 population differing by up to 7.5-fold across prefectures. The national estimated cost of IMCUs over the 2018–2022 period was ¥2,838 billion (mean ¥568 billion per year), approximately 1.5 times greater than total ICU cost (IMCU–ICU ratio ≈ 1.59).

Previous studies from high-income western countries over the past two decades have reported mean ICU costs per patient-day of €1230 (≒$1808) in Germany in 2008 [21], €1385 (≒$2037) in Italy in 2008 [21], €1,414 (≒$2077) in the Netherlands in 2008 [21], and €2025 (≒$2976) in the United Kingdom in 2008 [21], $4300 in the United States in 2010 [1, 2], €2,160 (≒$2549) in Belgium in 2018 [5], and AUD 4875 (≒$3389) in Australia in 2019 [6]. Against this backdrop, our findings indicate that the mean ICU cost per patient-day in Japan (¥197,277, ≒$1793) falls within the mid-to-lower range of reported values from Western countries.

The mean ICU cost per patient-day in Japan lies in the mid-to-lower range internationally, while the macroeconomic impact of ICU care—expressed as a proportion of GDP, OECD-defined total health expenditure, and OECD-defined hospital expenditures—is lower than any reported value in other high-income countries, including the United States, Canada, and Australia [2, 3, 6]. Notably, Japan allocates a relatively large share of its total health expenditure to hospital care (38.2%), indicating a hospital-centered health system. However, even within hospital spending, the proportion used for ICU services is substantially lower in Japan (1.42%) compared to the United States (13.3%), Canada (5.8%), and Australia (3.3%). When total health expenditure is considered as a percentage of GDP, Japan occupies an intermediate position among these countries (12.3%), suggesting adequate overall investment in health care. Nevertheless, the proportion of resources specifically allocated to intensive care remains modest (ICU costs account for only 0.065% of GDP). These findings suggest that although Japan spends a sufficient amount on health care overall, its allocation to critical care is low.

Relatively low ICU cost in Japan likely reflects strict fee schedule controls and the absence of national regulation for ICU bed establishment, resulting in limited capacity and significant regional disparities. The observed cost variation largely mirrors differences in ICU bed capacity across regions, suggesting that these disparities may be structurally driven rather than reflecting variations in efficiency or case-mix. Recent nationwide studies have shown that ICU bed capacity in Japan remains limited, and that ICU utilization and access to mechanical ventilation vary considerably by region, potentially influencing patient outcomes [22–24]. Emerging evidence further suggests that the current system may not sufficiently prioritize ICU admission for patients most likely to benefit, with some patients receiving critical care outside ICUs, a practice associated with worse outcomes in certain populations, such as those with pneumonia requiring mechanical ventilation or septic shock [25–27]. These findings highlight the need for Japan to reassess its investment in intensive care. Despite a rapidly aging population and an expected increase in critically ill patients [10], our analysis indicates that the macroeconomic impact of ICU care remained largely stable from 2018 to 2022, with minimal year-to-year variation, including during the COVID-19 pandemic [28]. This contrasts with trends in many Western countries, where the pandemic led to sharp increases in ICU spending and utilization [29, 30].

This study also found that IMCU costs in Japan were approximately 1.5 times greater than total ICU costs (IMCU–ICU cost ratio ≈ 1.59). This is the first national estimate of IMCU costs, as no previous research has quantified national-level IMCU costs. Although a previous US study did not directly report a national IMCU cost or IMCU–ICU cost ratio, the difference between the “moderate” and “conservative” national ICU cost estimates ($153.0 billion vs. $121.4 billion in 2008) [17] allows for an approximate calculation, suggesting an IMCU–ICU cost ratio of about 0.26. It is important to note that the US “moderate” estimate includes not only IMCU costs but also all other forms of post-ICU inpatient care prior to discharge, suggesting that the actual IMCU–ICU cost ratio in the US is likely even lower. Our findings, therefore, indicate that in Japan, a high proportion of national health care resources is allocated to IMCUs. As of 2022, Japan had 5.6 ICU beds and 14.7 IMCU beds per 100,000 population, approximately 2.5 times as many IMCU as ICU beds. Because Japan lacks regionally defined target numbers for ICU or IMCU beds and decisions regarding their establishment are left to individual hospitals, the lower staffing and cost requirements of IMCUs have likely facilitated their wider adoption, resulting in the observed 2.5-fold difference [11, 22, 23]. This may raise concerns, given that Japanese IMCUs generally lack intensivist staffing, operate with lower nurse-to-patient ratios of 1:3–1:5 than ICUs, and have been associated with higher mortality and readmission rates compared with ICUs [8, 9, 11, 25–27]. Importantly, this cost ratio should be interpreted primarily as reflecting structural and reimbursement-related differences in the organization and funding of IMCUs in Japan, rather than as direct evidence of inefficiency or inappropriate care.

In light of these findings, a careful reconsideration of how Japan allocates resources to intensive and intermediate care may be warranted. Although overall health spending is substantial, the proportion directed to ICU care appears relatively low. To promote equitable and sustainable critical care delivery, policymakers may consider expanding ICU capacity, setting national and regional ICU and IMCU bed targets, enhancing regional coordination to reduce disparities, providing financial or infrastructural support for hospitals seeking to establish new ICUs, and exploring new technologies, such as tele-ICU systems. Continued evaluation of the national cost of intensive care over time using similar standardized methods will also be essential to monitor trends and guide future policy decisions.

This study has several limitations. First, our cost estimates should be interpreted as claims-based proxies based on charges from the national fee schedule and administrative claims, rather than as actual expenditures (accounted costs) or final adjudicated or paid reimbursements under the DPC payment system. A previous Japanese single-center costing study estimated accounted costs of approximately ¥200,000 per patient-day (adjusted to 2022 purchasing power), which closely aligns with our charged cost estimates of ¥197,277 [7]. This concordance supports the validity of our costing approach. However, evidence from the same study also suggested that final reimbursements under the fee-for-service system represented about 85–90% of actual ICU costs [7]. Given the small magnitude of this difference, this potential underestimation is unlikely to have materially influenced our national estimates. Second, although we used a database covering about 70% of ICU beds nationwide as representative hospitals, participating hospitals tended to be larger and more often tertiary or academic centers. However, ICU-related characteristics were comparable between participating and non-participating hospitals, and the potential influence on national estimates cannot be fully excluded. Third, substantial variation exists in reported ICU costs both between and within countries, owing to differences in costing methodologies (e.g., hospital charges, claims data, or standardized tariffs), health-care financing structures, and the year in which estimates were derived [7–10]. For instance, US estimates frequently use the Russell equation (a top-down approach) with hospital charges or cost-to-charge ratios derived from large administrative databases [2], whereas European studies typically apply bottom-up approaches using actual expenditures or standardized fee schedules [7, 9]. Accounted-cost studies often show that personnel costs are the dominant driver of ICU costs [31]. Because our estimates are claim-based charges from Japan’s national fee schedule, such indirect costs are not itemized and may instead be absorbed into per-diem ICU management fees, which accounted for a particularly large share of ICU costs in our analysis. These methodological differences limit the direct comparability of ICU cost burdens across countries. Fourth, variation in ICU definition, organization, staffing, equipment, interventions, patient case-mix, and utilization of ICU varies between countries, influencing both costs and outcomes [5]. Although we aligned our ICU definitions with international standards, minor differences in unit classification or billing practices may still affect cross-country comparability [21, 32]. Furthermore, IMCU definitions are heterogeneous and lack international standardization [33], and cross-country comparisons should be interpreted cautiously. Fifth, as with all studies using large administrative data sets, coding errors or misclassification may have occurred. However, previous validation studies suggest high data accuracy in the DPC Study Group database [14]. Sixth, certain procedures that may be performed within ICUs, such as tracheotomy, might have been excluded, because their locations could not be clearly distinguished in the administrative data.

## Conclusion

This nationwide study presents the first comprehensive estimates of national ICU costs in Japan using robust, internationally comparable methods. The macroeconomic impact of ICU care in Japan is relatively low, indicating lower ICU spending than in other high-income countries. These findings highlight the need for Japan to reassess its investment and resource allocation in ICU care to ensure the efficient and equitable delivery of critical care services.

## Supplementary Information

Supplementary Material 1.

## Data Availability

The dataset analyzed in the current study is not publicly available because of contracts with the hospitals that provided data to the database.

## Abbreviations

CI: Confidence interval
DPC: Diagnosis procedure combination
GDP: Gross domestic product
ICU: Intensive care unit
IMCU: Intermediate care unit
IQR: Interquartile range
JPY: Japanese yen
SD: Standard deviation
