# Supplementary material for: National cost of intensive care in Japan from 2018 to 2022
Source: J Intensive Care. 2026 Mar 4;14:36. doi: 10.1186/s40560-026-00868-5 (PMC13069726; doi:10.1186/s40560-026-00868-5)
Supplement: Supplementary file 1 — Supplementary Material 1. [file 40560_2026_868_MOESM1_ESM.docx]

**Supplementary Materials**

**National Cost of Intensive Care in Japan from 2018 to 2022**

Hiroyuki Ohbe**^*^**, Daisuke Kudo, Shigeki Kushimoto, Yudai Iwasaki, Takuya Shiga, Rei Goto, Kei Nishiyama, Seo Ryutarou, Masao Iwagami, Toshikazu Abe, Yuya Kimura, Hiroki Matsui, Kiyohide Fushimi, Hideo Yasunaga

**^*^Corresponding author:**

Hiroyuki Ohbe

Department of Emergency and Critical Care Medicine, Tohoku University Hospital

1-1 Seiryo-machi, Aoba-ku, Sendai 980-8574, Japan

E-mail: [hohbey@gmail.com](mailto:hohbey@gmail.com); Tel: +81-2-2717-7489; Fax: +81-2-2717-7492

**List of Supplementary Materials**

**Supplementary Methods**

**Supplementary Table 1.** Japanese medical procedure codes used to define the ICU and IMCU in 2022

**Supplementary Table 2.** Characteristics of ICU and IMCU Costs by Management Fee Code in the DPC Study Group database from 2018 to 2022

**Supplementary Table 3.** Characteristics of ICU and IMCU by Management Fee Code in the Hospital Bed Function Report from 2018 to 2022

**Supplementary Table 4.** Comparison of Hospitals With and Without Participation in the DPC Study Group Database Among Hospitals With ICUs in 2022

**Supplementary Table 5.** Regional Variation in Estimated ICU, IMCU, and ICU+IMCU Costs per 100,000 Population in Japan’s 47 prefectures, 2018–2022

**Supplementary Figure 1.** Correlation between Regional ICU and IMCU Cost Estimates and Corresponding Bed Densities in Japan

**Supplementary Methods**

This appendix provides the full technical details of the data structure, variable definitions, cost aggregation algorithms, and analytic procedures. The main manuscript includes only a concise methodological summary.

**Methods**

***Study design and data source***

We conducted a nationwide, retrospective cohort study using two data sources: the Diagnosis Procedure Combination (DPC) Study Group database and the Hospital Bed Function Report from fiscal year 2018–2022 [12,13].

The DPC Study Group database includes discharge abstract data (Format 1), administrative claims data (EF files), and hospital-level data (Format 3) from more than 1,500 voluntarily participating acute-care hospitals, representing over 50% of acute-care hospital beds nationwide [12]. Format 1 contains admission-level demographic and clinical variables, such as age, sex, diagnoses recorded using the International Classification of Diseases, Tenth Revision codes, and discharge status. The EF files provide date-stamped records of all surgical, pharmaceutical, laboratory, and other inpatient services. They also include the estimated total cost of each service, calculated according to the reference prices specified in Japan’s national fee schedule for all medical services and products. Format 3 includes hospital-level data, including the number of beds in each ward, the inpatient fee billed per ward, and the assigned ward codes for each fiscal year. By linking EF files with Format 3, we identified, for each day, the type of care unit, such as ICU or IMCU, corresponding to each ward code. A previous validation study of this database revealed that the sensitivity and specificity of EF file procedures exceeded 90% each [14].

We also used the Hospital Bed Function Report, published annually by the Ministry of Health, Labour, and Welfare of Japan, which provides functional and statistical information on hospitals as of July 1 each year [13]. The report includes hospital type (e.g., academic or tertiary emergency hospital), ward classification (e.g., general ward, ICU, or IMCU), the number of licensed hospital beds by ward type, and the annual volumes of patients and patient-days by ward type.

This study was approved by the Institutional Review Board of the University of Tokyo (approval number: 3501-5; approval date: May 19, 2021). Because all data were de-identified, the requirement for informed consent was waived. This study adhered to the principles of the Declaration of Helsinki.

***ICU Cost Estimation Framework***

To estimate the national ICU costs in Japan, we applied a bottom-up costing approach based on the national projection method described by Halpern & Pastores [15]. An overview of this approach is shown in **Figure 1**. The national projection method estimates ICU costs by aggregating detailed, itemized cost data from a representative sample of hospitals and summing all billed services for each ICU day to calculate patient-day level ICU costs. These patient-day level costs are then extrapolated to national estimates by weighting them according to the annual number of ICU bed-days, thereby providing a granular and accurate assessment of ICU resource use and cost. This approach contrasts with the traditional Russell equation (top-down method) [1,15], which relies on aggregated average costs and fixed ICU-to-non-ICU cost ratios from national datasets to generate population-level estimates.

The analytic perspective of this study was that of the payer, with the objective of informing policy evaluation and resource allocation. In Japan, health care expenditures are financed through a combination of insurance premiums, taxes, and patient co-payments. Accordingly, we used charged costs based on reference prices in Japan’s national fee schedule and derived from administrative claims data as our primary cost measure. These most accurately reflect expenditures from the payer’s perspective and are routinely used for policy planning in the Japanese health system. Accounted (actual) costs, representing the total costs incurred by hospitals, including indirect costs such as personnel, utilities, depreciation, and overhead, were not directly available. However, because Japan’s national fee schedule is periodically revised, the charged costs closely approximate the accounted costs incurred by hospitals [16].

***Definition of ICU Cost and Cost Calculation***

The primary outcome of this study was the national ICU cost, estimated using definitions and methods consistent with those in major previous ICU cost studies [1,2,17]. In this study, an ICU was defined as an adult or pediatric unit formally certified by the Japanese Society of Intensive Care Medicine, defined as a separate unit providing critical care services with at least one physician on site 24 h per day, a minimum of two intensivists, around-the-clock nursing with a nurse-to-patient ratio of >1:2, an ICU nurse, a clinical engineer present in the hospital 24 h per day, and equipment necessary for the management of critically ill patients [18]. Costs of IMCUs—also known as high-dependency units, high-care units, or step-down units—were excluded. Additional details and the procedure codes used to define the ICUs are provided in **Supplementary Table 1**. When calculating ICU costs, we excluded billed charges for surgery and cardiac catheterization, as these procedures were assumed to have been performed in the operating room or catheterization laboratory rather than the ICU.

All inpatient medical costs were calculated using the variable ‘EF-16: Achieved fee-for-service points’ from the integrated EF file of the DPC database. This variable represents the total cost of all medical services provided during hospitalization, including both fee-for-service items and the equivalent point values of bundled services. We did not incorporate institution-specific coefficients (recorded in the D file) because they apply to bundled reimbursement and cannot be allocated to ICU cost calculations.

ICU-designated wards were identified using ward codes recorded in Format 3. These codes were then used to identify ICU-designated days and to extract all billed services from the EF files for each ICU-designated ward. This approach defines ICU-designated days based on the ward location rather than billing codes. Thus, even when ICU stays exceeded the reimbursement limit (e.g., 14 days), those days were still regarded as ICU-designated days and included in the cost aggregation. Data from wards that could not be linked to Format 3 were excluded from the analysis. ICU costs were aggregated at the ICU management fee code and fiscal year level by summing “EF-16: Achieved fee-for-service points” for all billed services on ICU-designated days in the EF files, after excluding the surgical and catheterization costs as described above. In practice, these excluded costs were identified by matching administrative records that shared a common sequence number with the corresponding anesthesia (Japanese medical procedure code: L008–L010), surgical billing codes (K000–K915, excluding the maintenance fee for mechanical circulatory support), and cardiac catheterization code (D206) in the EF files. The mean ICU cost per patient-day was calculated by dividing the total cost by the total number of patient-days for each ICU management fee code and fiscal year. National ICU costs were then estimated by multiplying the average cost per patient-day for each ICU management fee code and fiscal year by the annual volume of patient-days for that code, as reported in the Hospital Bed Function Report [13], yielding total annual national ICU costs. ICU and IMCU costs were first estimated in nominal terms. We then derived real (inflation-adjusted) costs using the official biennial revision rates of the national fee schedule (+0.55% in 2020 and +0.43% in 2022).

***Macroeconomic Impact, Regional Variation, and Detailed Cost Breakdown***

We evaluated national ICU costs as a proportion of key macroeconomic and health expenditure indicators for each year. These indicators included nominal GDP, OECD-defined total health expenditure, OECD-defined hospital expenditures, and national inpatient expenditure as defined by the Ministry of Health, Labour and Welfare of Japan. These proportions allow evaluation of the relative financial priority assigned to intensive care in the context of national economic capacity (GDP), overall health care spending (total health expenditure), and hospital resource allocation (hospital and inpatient expenditures). Data for these indicators were obtained from OECD Health Statistics [19] and official Japanese government sources [20]. When comparable national ICU cost data from other countries were available from published studies, we derived their nominal GDP, OECD-defined total health expenditure, and OECD-defined hospital expenditures from OECD Health Statistics [18] to enable cross-country comparison of relative financial priorities.

We also examined regional variation by estimating ICU costs per 100,000 population for each of Japan’s 47 prefectures during 2018–2022. Prefecture-specific ICU costs were visualized using a choropleth map to illustrate geographic differences. We then assessed the correlation between prefecture-specific ICU costs and ICU bed density (beds per 100,000 population) using linear regression analysis and Pearson’s correlation coefficients.

To examine the composition of ICU costs, we conducted two types of cost breakdowns for the period 2018–2022. First, ICU costs were disaggregated into 10 specific categories—consultation (classification code: 11–14), oral drugs (21–28), injections (31–39), procedures (40), transfusion and mechanical circulatory support (50 and 54), tests (60), radiology (70), inpatient fees (90, 92), diet (97), and other services (80)—based on classification code (*kubun* codes) defined in Japan’s national fee schedule. Second, ICU costs were disaggregated into each ICU management fee code, and the percentage contribution of each fee code to total costs was calculated.

***Analysis of IMCU Costs***

We also estimated national IMCU costs. The definition of IMCU in Japan is similar to that of the ICU, except that IMCUs are not required to have intensivist staffing or an ICU nurse, and the nurse-to-patient ratio is 1:3, 1:4, or 1:5. Additional details are provided in **Supplementary Table 1**. The same costing and aggregation procedures described above were applied. We also calculated combined national estimates for ICU and IMCU costs, and evaluated their macroeconomic impact, regional variation, and detailed cost breakdown.

***Statistical analyses***

Continuous variables were summarized as means with standard deviation (SD) or medians with interquartile ranges (IQRs), as appropriate. Categorical variables were expressed as frequencies and percentages. To assess temporal trends, we performed linear regression analyses treating fiscal year as a continuous independent variable and reported the p for trend based on the regression coefficient. A two-sided P value < 0.05 was considered statistically significant. All analyses were performed using Stata/SE, Version 19.0 (StataCorp, College Station, TX, USA).

**Supplementary Table 1.** Japanese Medical Procedure Codes Used to Define ICUs and IMCUs in 2022

|  |  |  | Cost | Nurse to |  | Non- | Physician | Certified | Clinical | Reimbursable |
| --- | --- | --- | --- | --- | --- | --- | --- | --- | --- | --- |
|  |  |  | per day, | patient | Intensivist | intensivist | night | ICU | Engineer | Duration, |
| Type | Code | Description | yen | ratio | staffing | Staffing | shift | Nurse | **** | days |
| ICU | A3011 | ICU management fee 1 | 142,110** | 1:2 | ≥2 in ICU 24/7 | – | Dedicated to ICU | Required  *** | 24/7 in hospital | 14 |
| ICU | A3012 | ICU management fee 2 | 142,110** | 1:2 | ≥2 in ICU 24/7 | – | Dedicated to ICU | Required  *** | 24/7 in hospital | 14  ***** |
| ICU | A3013 | ICU management fee 3 | 96,970** | 1:2 | – | ≥1 in ICU 24/7 | Dedicated to ICU | – | – | 14 |
| ICU | A3014 | ICU management fee 4 | 96,970** | 1:2 | – | ≥1 in ICU 24/7 | Dedicated to ICU | – | – | 14  ***** |
| ICU | A3002 | Emergency and critical care unit management fee 2 | 118,020* | 1:2 | – | ≥1 in ICU 24/7 | Dedicated to ICU | – | – | 14 |
| ICU | A3004 | Emergency and critical care unit management fee 4 | 118,020* | 1:2 | – | ≥1 in ICU 24/7 | Dedicated to ICU | – | – | 14  ***** |
| ICU | A301-4 | Pediatric ICU management fee | 163,170** | 1:2 | ≥2 in ICU 24/7 | – | Dedicated to ICU | – | – | 14  ****** |
| IMCU | A3001 | Emergency and critical care unit management fee 1 | 102,230* | 1:4 | – | ≥1 in IMCU 24/7 | Dedicated to IMCU | – | – | 14 |
| IMCU | A3003 | Emergency and critical care unit management fee 3 | 102,230* | 1:4 | – | ≥1 in IMCU 24/7 | Dedicated to IMCU | – | – | 14  ***** |
| IMCU | A301-21 | High care unit management fee 1 | 68,550 | 1:4 | – | ≥1 in hospital 24/7 | On-call permit | – | – | 21 |
| IMCU | A301-22 | High care unit management fee 2 | 42,240 | 1:5 | – | ≥1 in hospital 24/7 | On-call permit | – | – | 21 |
| IMCU | A301-3 | Stroke care unit management fee | 60,130 | 1:3 | – | Neurologist ≥1 in hospital 24/7 | On-call permit | – | – | 14 |
| IMCU | A3021 | Neonatal ICU management fee 1 | 105,390 | 1:3 | – | ≥1 in IMCU 24/7 | Dedicated to IMCU | – | – | 21  ****** |
| IMCU | A3022 | Neonatal ICU management fee 2 | 84,340 | 1:3 | – | ≥1 in IMCU 24/7 | Dedicated to IMCU | – | – | 21  ****** |
| IMCU | A3031 | Perinatal Maternal-Fetal ICU management fee | 73,810 | 1:3 | – | ≥1 in IMCU 24/7,  Obstetrician ≥2 in hospital 24/7 | Dedicated to IMCU | – | – | 14 |
| IMCU | A3032 | Perinatal Neonatal ICU management fee | 105,390 | 1:3 | – | ≥1 in IMCU 24/7,  Obstetrician ≥2 in hospital 24/7 | Dedicated to IMCU | – | – | 21  ****** |

*Cost per day for the first 3 days after admission in 2022.

**Cost per day for the first 7 days after admission in 2022.

***An ICU nurse is defined as a full-time, dedicated nurse with at least five years of experience in caring for critically ill patients and completion of appropriate training, assigned to the ICU for at least 20 hours per week.

****A clinical engineer is defined as a full-time, dedicated allied health professional responsible for the operation and maintenance of life-support equipment and is present in the hospital 24 hours a day.

*****The reimbursable duration is extended to 60 days for extensive burns.

******The maximum reimbursable duration is extended to 21 days for patients requiring acute blood purification (except peritoneal dialysis), high-risk cardiac surgery, hypoplastic left heart syndrome, acute respiratory distress syndrome, or myocarditis/cardiomyopathy; up to 35 days for patients requiring extracorporeal membrane oxygenation; and up to 55 days for neonates undergoing surgery for congenital heart disease.

*******The maximum reimbursable duration is 35 days for neonates ≥1,500g with designated primary diseases, 60 days for neonates 1,000–1,500g, 90 days for neonates <1,000g, 105 days for neonates 500–750g with chronic lung disease, and 110 days for neonates <500g with chronic lung disease.

ICU, intensive care unit; IMCU, intermediate care unit

**Supplementary Table 2.** Characteristics of ICU and IMCU by Management Fee Code in the DPC Study Group database from 2018 to 2022

|  |  |  |  |  | Number |  | Mean | Mean |
| --- | --- | --- | --- | --- | --- | --- | --- | --- |
|  |  | Number | Number | Number | of | Total | cost per | cost per |
|  | Fiscal | of | of | of | patient | cost, | patient, | patient day, |
| Description | year | hospital | beds | Patients | days | yen | yen | yen |
| **ICU** |  |  |  |  |  |  |  |  |
| A3011: ICU management fee 1 | 2018 | 67 | 799 | 51296 | 200234 | 40814349869 | 795663 | 203833 |
| A3012: ICU management fee 2 | 2018 | 30 | 307 | 19346 | 72336 | 15364960251 | 794219 | 212411 |
| A3013: ICU management fee 3 | 2018 | 184 | 1556 | 102829 | 391016 | 62464300810 | 607458 | 159749 |
| A3014: ICU management fee 4 | 2018 | 51 | 548 | 35968 | 128263 | 22513299790 | 625926 | 175525 |
| A3002: Emergency and Critical care unit management fee 2 | 2018 | 12 | 104 | 5545 | 23410 | 3967978223 | 715596 | 169499 |
| A3004: Emergency and Critical care unit management fee 4 | 2018 | 54 | 664 | 35565 | 175149 | 31883888343 | 896496 | 182039 |
| A301-4: Pediatric ICU management fee | 2018 | 4 | 44 | 1963 | 11472 | 2394601410 | 1219868 | 208734 |
| A3011: ICU management fee 1 | 2019 | 98 | 1169 | 78530 | 303342 | 63807421411 | 812523 | 210348 |
| A3012: ICU management fee 2 | 2019 | 54 | 687 | 40586 | 156893 | 35331988953 | 870546 | 225198 |
| A3013: ICU management fee 3 | 2019 | 189 | 1602 | 105491 | 400713 | 63249766612 | 599575 | 157843 |
| A3014: ICU management fee 4 | 2019 | 56 | 605 | 40568 | 150032 | 27290531482 | 672711 | 181898 |
| A3002: Emergency and Critical care unit management fee 2 | 2019 | 16 | 142 | 8103 | 35583 | 5830638655 | 719565 | 163860 |
| A3004: Emergency and Critical care unit management fee 4 | 2019 | 63 | 746 | 42021 | 204503 | 37226054544 | 885892 | 182032 |
| A301-4: Pediatric ICU management fee | 2019 | 4 | 52 | 1942 | 12227 | 2443565469 | 1258273 | 199850 |
| A3011: ICU management fee 1 | 2020 | 98 | 1170 | 72102 | 282164 | 63396005558 | 879254 | 224678 |
| A3012: ICU management fee 2 | 2020 | 58 | 736 | 43470 | 165965 | 41495094433 | 954569 | 250023 |
| A3013: ICU management fee 3 | 2020 | 188 | 1569 | 102780 | 386117 | 62943027957 | 612405 | 163015 |
| A3014: ICU management fee 4 | 2020 | 54 | 575 | 34272 | 125482 | 23625454833 | 689352 | 188278 |
| A3002: Emergency and Critical care unit management fee 2 | 2020 | 17 | 142 | 7631 | 27708 | 5090077727 | 667026 | 183704 |
| A3004: Emergency and Critical care unit management fee 4 | 2020 | 60 | 750 | 37286 | 172044 | 35049428058 | 940016 | 203724 |
| A301-4: Pediatric ICU management fee | 2020 | 4 | 52 | 1553 | 9878 | 2059530872 | 1326163 | 208497 |
| A3011: ICU management fee 1 | 2021 | 107 | 1341 | 75424 | 291886 | 68254584888 | 904945 | 233840 |
| A3012: ICU management fee 2 | 2021 | 67 | 872 | 48615 | 192595 | 49796733148 | 1024308 | 258557 |
| A3013: ICU management fee 3 | 2021 | 188 | 1635 | 100988 | 384059 | 63237561740 | 626189 | 164656 |
| A3014: ICU management fee 4 | 2021 | 49 | 534 | 31252 | 117609 | 22864932867 | 731631 | 194415 |
| A3002: Emergency and Critical care unit management fee 2 | 2021 | 18 | 150 | 8766 | 32241 | 6911252980 | 788416 | 214362 |
| A3004: Emergency and Critical care unit management fee 4 | 2021 | 59 | 823 | 36416 | 174399 | 38690807827 | 1062467 | 221852 |
| A301-4: Pediatric ICU management fee | 2021 | 5 | 64 | 1424 | 7909 | 1598962953 | 1122867 | 202170 |
| A3011: ICU management fee 1 | 2022 | 100 | 1189 | 69879 | 255397 | 59439200816 | 850602 | 232733 |
| A3012: ICU management fee 2 | 2022 | 69 | 868 | 46901 | 174768 | 45065729535 | 960869 | 257860 |
| A3013: ICU management fee 3 | 2022 | 167 | 1440 | 92835 | 326998 | 55981293464 | 603019 | 171198 |
| A3014: ICU management fee 4 | 2022 | 42 | 451 | 26395 | 95025 | 18292754023 | 693039 | 192505 |
| A3002: Emergency and Critical care unit management fee 2 | 2022 | 15 | 128 | 8628 | 30133 | 6050751075 | 701292 | 200801 |
| A3004: Emergency and Critical care unit management fee 4 | 2022 | 58 | 711 | 36131 | 159116 | 35381551472 | 979257 | 222363 |
| A301-4: Pediatric ICU management fee | 2022 | 5 | 53 | 1428 | 9022 | 1845008378 | 1292023 | 204501 |
| **IMCU** |  |  |  |  |  |  |  |  |
| A3001: Emergency and Critical care unit management fee 1 | 2018 | 108 | 2216 | 102098 | 416768 | 57783394526 | 565960 | 138646 |
| A3003: Emergency and Critical care unit management fee 3 | 2018 | 45 | 1048 | 43223 | 174780 | 25531652326 | 590696 | 146079 |
| A301-21: High care unit management fee 1 | 2018 | 287 | 2968 | 188652 | 885678 | 84778320739 | 449390 | 95721 |
| A301-22: High care unit management fee 2 | 2018 | 18 | 187 | 9362 | 51926 | 4026449951 | 430084 | 77542 |
| A301-3: Stroke care unit management fee | 2018 | 89 | 802 | 32506 | 293438 | 28171412663 | 866653 | 96005 |
| A3021: Neonatal ICU management fee 1 | 2018 | 42 | 389 | 6185 | 119298 | 14819319592 | 2396010 | 124221 |
| A3022: Neonatal ICU management fee 2 | 2018 | 74 | 472 | 8470 | 98358 | 10740377601 | 1268049 | 109197 |
| A3031: Perinatal Maternal-Fetal ICU management fee | 2018 | 71 | 524 | 14326 | 178547 | 15035918743 | 1049555 | 84213 |
| A3032: Perinatal Neonatal ICU management fee | 2018 | 64 | 980 | 13971 | 298626 | 36141239395 | 2586876 | 121025 |
| A3001: Emergency and Critical care unit management fee 1 | 2019 | 129 | 2549 | 119481 | 492029 | 68658378671 | 574638 | 139541 |
| A3003: Emergency and Critical care unit management fee 3 | 2019 | 62 | 1400 | 64804 | 267937 | 39523565042 | 609894 | 147511 |
| A301-21: High care unit management fee 1 | 2019 | 317 | 3288 | 223290 | 961716 | 95058276673 | 425717 | 98842 |
| A301-22: High care unit management fee 2 | 2019 | 19 | 177 | 8572 | 50734 | 4186070138 | 488342 | 82510 |
| A301-3: Stroke care unit management fee | 2019 | 96 | 813 | 34376 | 315562 | 30149249822 | 877044 | 95541 |
| A3021: Neonatal ICU management fee 1 | 2019 | 57 | 538 | 8277 | 157892 | 19330885921 | 2335494 | 122431 |
| A3022: Neonatal ICU management fee 2 | 2019 | 90 | 512 | 10358 | 126329 | 14069551985 | 1358327 | 111372 |
| A3031: Perinatal Maternal-Fetal ICU management fee | 2019 | 106 | 710 | 19771 | 237680 | 20202269023 | 1021813 | 84998 |
| A3032: Perinatal Neonatal ICU management fee | 2019 | 96 | 1356 | 19034 | 402231 | 50379845758 | 2646835 | 125251 |
| A3001: Emergency and Critical care unit management fee 1 | 2020 | 123 | 2441 | 105309 | 427999 | 66409406355 | 630615 | 155163 |
| A3003: Emergency and Critical care unit management fee 3 | 2020 | 65 | 1412 | 60343 | 245336 | 38984747453 | 646053 | 158904 |
| A301-21: High care unit management fee 1 | 2020 | 342 | 3713 | 223284 | 975029 | 102179037445 | 457619 | 104796 |
| A301-22: High care unit management fee 2 | 2020 | 20 | 199 | 9157 | 39298 | 3783283633 | 413158 | 96272 |
| A301-3: Stroke care unit management fee | 2020 | 102 | 879 | 35959 | 335956 | 33487460912 | 931268 | 99678 |
| A3021: Neonatal ICU management fee 1 | 2020 | 58 | 548 | 9070 | 166405 | 21537877254 | 2374628 | 129430 |
| A3022: Neonatal ICU management fee 2 | 2020 | 89 | 513 | 10161 | 122776 | 13996411420 | 1377464 | 114000 |
| A3031: Perinatal Maternal-Fetal ICU management fee | 2020 | 105 | 692 | 19775 | 220167 | 19672198575 | 994801 | 89351 |
| A3032: Perinatal Neonatal ICU management fee | 2020 | 94 | 1369 | 20385 | 421256 | 55063636937 | 2701184 | 130713 |
| A3001: Emergency and Critical care unit management fee 1 | 2021 | 130 | 2629 | 113766 | 444445 | 73490752331 | 645982 | 165354 |
| A3003: Emergency and Critical care unit management fee 3 | 2021 | 63 | 1369 | 57055 | 233414 | 39936081721 | 699958 | 171095 |
| A301-21: High care unit management fee 1 | 2021 | 356 | 3922 | 229632 | 1003571 | 111089843433 | 483773 | 110695 |
| A301-22: High care unit management fee 2 | 2021 | 23 | 233 | 10599 | 49274 | 4752091976 | 448353 | 96442 |
| A301-3: Stroke care unit management fee | 2021 | 114 | 987 | 38719 | 356989 | 36394676879 | 939969 | 101949 |
| A3021: Neonatal ICU management fee 1 | 2021 | 59 | 576 | 8831 | 157944 | 20398276143 | 2309849 | 129149 |
| A3022: Neonatal ICU management fee 2 | 2021 | 88 | 528 | 10461 | 127080 | 14368162489 | 1373498 | 113064 |
| A3031: Perinatal Maternal-Fetal ICU management fee | 2021 | 106 | 716 | 20117 | 221551 | 20427673695 | 1015443 | 92203 |
| A3032: Perinatal Neonatal ICU management fee | 2021 | 97 | 1458 | 21335 | 424920 | 55221267646 | 2588295 | 129957 |
| A3001: Emergency and Critical care unit management fee 1 | 2022 | 120 | 2404 | 111551 | 429595 | 71953294094 | 645026 | 167491 |
| A3003: Emergency and Critical care unit management fee 3 | 2022 | 62 | 1298 | 59497 | 234786 | 39479202820 | 663550 | 168150 |
| A301-21: High care unit management fee 1 | 2022 | 317 | 3453 | 204473 | 856244 | 96726470626 | 473053 | 112966 |
| A301-22: High care unit management fee 2 | 2022 | 21 | 211 | 11312 | 49021 | 4929679054 | 435792 | 100563 |
| A301-3: Stroke care unit management fee | 2022 | 113 | 986 | 35206 | 328946 | 34452835670 | 978607 | 104737 |
| A3021: Neonatal ICU management fee 1 | 2022 | 53 | 516 | 8275 | 132878 | 17877908134 | 2160472 | 134544 |
| A3022: Neonatal ICU management fee 2 | 2022 | 83 | 495 | 9790 | 115149 | 13317135948 | 1360280 | 115651 |
| A3031: Perinatal Maternal-Fetal ICU management fee | 2022 | 95 | 652 | 17524 | 185063 | 17774777707 | 1014311 | 96047 |
| A3032: Perinatal Neonatal ICU management fee | 2022 | 84 | 1233 | 16834 | 309010 | 41638645131 | 2473485 | 134749 |

ICU, intensive care unit; IMCU, intermediate care unit

**Supplementary Table 3.** Characteristics of ICU and IMCU by Management Fee Code in the Hospital Bed Function Report from 2018 to 2022

|  |  |  |  |  |  | Covering rate |
| --- | --- | --- | --- | --- | --- | --- |
|  |  |  |  |  |  | of critical |
|  |  |  | Number |  | Number | care beds |
|  |  | Number | of | Number | of | in DPC |
|  | Fiscal | of | ICU | of | patient | database, |
| Description | year | hospital | beds | patients | days | % |
| **ICU** |  |  |  |  |  |  |
| A3011: ICU management fee 1 | 2018 | 126 | 1331 | 89438 | 346468 | 60.0 |
| A3012: ICU management fee 2 | 2018 | 64 | 698 | 43432 | 177049 | 44.0 |
| A3013: ICU management fee 3 | 2018 | 396 | 3045 | 209201 | 782501 | 51.1 |
| A3014: ICU management fee 4 | 2018 | 81 | 787 | 53682 | 217977 | 69.6 |
| A3002: Emergency and Critical care unit management fee 2 | 2018 | 35 | 311 | 18664 | 79409 | 33.4 |
| A3004: Emergency and Critical care unit management fee 4 | 2018 | 83 | 887 | 54666 | 254220 | 74.9 |
| A301-4: Pediatric ICU management fee | 2018 | 9 | 145 | 3421 | 35107 | 30.3 |
| A3011: ICU management fee 1 | 2019 | 140 | 1483 | 99957 | 427675 | 78.8 |
| A3012: ICU management fee 2 | 2019 | 69 | 749 | 51235 | 197397 | 91.7 |
| A3013: ICU management fee 3 | 2019 | 376 | 2892 | 198980 | 725636 | 55.4 |
| A3014: ICU management fee 4 | 2019 | 74 | 748 | 48855 | 198562 | 80.9 |
| A3002: Emergency and Critical care unit management fee 2 | 2019 | 30 | 259 | 15554 | 94318 | 54.8 |
| A3004: Emergency and Critical care unit management fee 4 | 2019 | 82 | 899 | 55661 | 262618 | 83.0 |
| A301-4: Pediatric ICU management fee | 2019 | 8 | 104 | 7003 | 22456 | 50.0 |
| A3011: ICU management fee 1 | 2020 | 149 | 1552 | 112005 | 411972 | 75.4 |
| A3012: ICU management fee 2 | 2020 | 76 | 847 | 59241 | 224903 | 86.9 |
| A3013: ICU management fee 3 | 2020 | 355 | 2727 | 189079 | 674037 | 57.5 |
| A3014: ICU management fee 4 | 2020 | 67 | 688 | 46759 | 173776 | 83.6 |
| A3002: Emergency and Critical care unit management fee 2 | 2020 | 25 | 208 | 13958 | 53896 | 68.3 |
| A3004: Emergency and Critical care unit management fee 4 | 2020 | 80 | 889 | 53797 | 249347 | 84.4 |
| A301-4: Pediatric ICU management fee | 2020 | 8 | 104 | 3375 | 25246 | 50.0 |
| A3011: ICU management fee 1 | 2021 | 161 | 1674 | 106112 | 425206 | 80.1 |
| A3012: ICU management fee 2 | 2021 | 81 | 919 | 57970 | 220309 | 94.9 |
| A3013: ICU management fee 3 | 2021 | 354 | 2757 | 177592 | 638636 | 59.3 |
| A3014: ICU management fee 4 | 2021 | 61 | 622 | 37750 | 146060 | 85.9 |
| A3002: Emergency and Critical care unit management fee 2 | 2021 | 27 | 218 | 14923 | 45142 | 68.8 |
| A3004: Emergency and Critical care unit management fee 4 | 2021 | 81 | 909 | 49330 | 230067 | 90.5 |
| A301-4: Pediatric ICU management fee | 2021 | 9 | 122 | 3831 | 26682 | 52.5 |
| A3011: ICU management fee 1 | 2022 | 159 | 1681 | 106649 | 426167 | 70.7 |
| A3012: ICU management fee 2 | 2022 | 81 | 963 | 60820 | 327611 | 90.1 |
| A3013: ICU management fee 3 | 2022 | 324 | 2491 | 166700 | 593512 | 57.8 |
| A3014: ICU management fee 4 | 2022 | 56 | 592 | 35136 | 135533 | 76.2 |
| A3002: Emergency and Critical care unit management fee 2 | 2022 | 20 | 157 | 11325 | 37318 | 81.5 |
| A3004: Emergency and Critical care unit management fee 4 | 2022 | 81 | 921 | 48618 | 232027 | 77.2 |
| A301-4: Pediatric ICU management fee | 2022 | 16 | 207 | 6274 | 49025 | 25.6 |
| **IMCU** |  |  |  |  |  |  |
| A3001: Emergency and Critical care unit management fee 1 | 2018 | 197 | 3378 | 243902 | 881412 | 65.6 |
| A3003: Emergency and Critical care unit management fee 3 | 2018 | 89 | 1733 | 123334 | 484806 | 60.5 |
| A301-21: High care unit management fee 1 | 2018 | 593 | 5000 | 353330 | 1249801 | 59.4 |
| A301-22: High care unit management fee 2 | 2018 | 47 | 455 | 25665 | 105834 | 41.1 |
| A301-3: Stroke care unit management fee | 2018 | 167 | 1368 | 56239 | 437177 | 58.6 |
| A3021: Neonatal ICU management fee 1 | 2018 | 89 | 951 | 23710 | 279502 | 40.9 |
| A3022: Neonatal ICU management fee 2 | 2018 | 143 | 867 | 20374 | 231578 | 54.4 |
| A3031: Perinatal Maternal-Fetal ICU management fee | 2018 | 123 | 846 | 27924 | 243324 | 61.9 |
| A3032: Perinatal Neonatal ICU management fee | 2018 | 109 | 1565 | 26502 | 483255 | 62.6 |
| A3001: Emergency and Critical care unit management fee 1 | 2019 | 204 | 3641 | 262168 | 999267 | 70.0 |
| A3003: Emergency and Critical care unit management fee 3 | 2019 | 85 | 1733 | 116266 | 485842 | 80.8 |
| A301-21: High care unit management fee 1 | 2019 | 651 | 5533 | 391995 | 1392191 | 59.4 |
| A301-22: High care unit management fee 2 | 2019 | 32 | 336 | 18643 | 75656 | 52.7 |
| A301-3: Stroke care unit management fee | 2019 | 177 | 1384 | 59238 | 442407 | 58.7 |
| A3021: Neonatal ICU management fee 1 | 2019 | 80 | 762 | 13968 | 253525 | 70.6 |
| A3022: Neonatal ICU management fee 2 | 2019 | 146 | 872 | 20202 | 233045 | 58.7 |
| A3031: Perinatal Maternal-Fetal ICU management fee | 2019 | 124 | 832 | 30078 | 257760 | 85.3 |
| A3032: Perinatal Neonatal ICU management fee | 2019 | 115 | 1653 | 26763 | 523480 | 82.0 |
| A3001: Emergency and Critical care unit management fee 1 | 2020 | 196 | 3488 | 244133 | 926069 | 70.0 |
| A3003: Emergency and Critical care unit management fee 3 | 2020 | 85 | 1717 | 116761 | 454209 | 82.2 |
| A301-21: High care unit management fee 1 | 2020 | 682 | 6001 | 399803 | 1489710 | 61.9 |
| A301-22: High care unit management fee 2 | 2020 | 36 | 381 | 20599 | 98320 | 52.2 |
| A301-3: Stroke care unit management fee | 2020 | 177 | 1416 | 59956 | 474560 | 62.1 |
| A3021: Neonatal ICU management fee 1 | 2020 | 83 | 809 | 13432 | 678842 | 67.7 |
| A3022: Neonatal ICU management fee 2 | 2020 | 144 | 885 | 19126 | 222405 | 58.0 |
| A3031: Perinatal Maternal-Fetal ICU management fee | 2020 | 124 | 809 | 26221 | 229780 | 85.5 |
| A3032: Perinatal Neonatal ICU management fee | 2020 | 113 | 1642 | 26798 | 515246 | 83.4 |
| A3001: Emergency and Critical care unit management fee 1 | 2021 | 199 | 3564 | 217328 | 809128 | 73.8 |
| A3003: Emergency and Critical care unit management fee 3 | 2021 | 88 | 1719 | 104178 | 411066 | 79.6 |
| A301-21: High care unit management fee 1 | 2021 | 765 | 7432 | 417759 | 1625149 | 52.8 |
| A301-22: High care unit management fee 2 | 2021 | 43 | 558 | 24989 | 109393 | 41.8 |
| A301-3: Stroke care unit management fee | 2021 | 188 | 1518 | 60773 | 469305 | 65.0 |
| A3021: Neonatal ICU management fee 1 | 2021 | 80 | 785 | 12771 | 222025 | 73.4 |
| A3022: Neonatal ICU management fee 2 | 2021 | 140 | 868 | 18336 | 222422 | 60.8 |
| A3031: Perinatal Maternal-Fetal ICU management fee | 2021 | 131 | 856 | 27083 | 225807 | 83.6 |
| A3032: Perinatal Neonatal ICU management fee | 2021 | 115 | 1693 | 25662 | 508111 | 86.1 |
| A3001: Emergency and Critical care unit management fee 1 | 2022 | 198 | 3615 | 220020 | 777537 | 66.5 |
| A3003: Emergency and Critical care unit management fee 3 | 2022 | 74 | 1480 | 95946 | 370852 | 87.7 |
| A301-21: High care unit management fee 1 | 2022 | 741 | 6897 | 425328 | 1554695 | 50.1 |
| A301-22: High care unit management fee 2 | 2022 | 56 | 665 | 29662 | 130398 | 31.7 |
| A301-3: Stroke care unit management fee | 2022 | 187 | 1528 | 60617 | 494748 | 64.5 |
| A3021: Neonatal ICU management fee 1 | 2022 | 77 | 773 | 12465 | 218820 | 66.8 |
| A3022: Neonatal ICU management fee 2 | 2022 | 144 | 909 | 20913 | 247879 | 54.5 |
| A3031: Perinatal Maternal-Fetal ICU management fee | 2022 | 123 | 809 | 28329 | 224336 | 80.6 |
| A3032: Perinatal Neonatal ICU management fee | 2022 | 113 | 1669 | 27748 | 510739 | 73.9 |

ICU, intensive care unit; IMCU, intermediate care unit

**Supplementary Table 4.** Comparison of Hospitals With and Without Participation in the DPC Study Group Database Among Hospitals With ICUs in 2022

|  |  | Non-DPC-participating | DPC-participating |
| --- | --- | --- | --- |
|  | Total | hospitals with ICUs | hospitals with ICUs |
|  | N=593 | N=244 | N=349 |
| Number of total inpatient beds | 449 (346-592) | 382 (283-470) | 520 (397-651) |
| Academic hospital | 81 (13.7) | 8 (3.3) | 73 (20.9) |
| Tertiary emergency hospital | 262 (44.2) | 73 (29.9) | 189 (54.2) |
| Annual volume of ambulance acceptance | 3777 (2524-5516) | 3494 (2289-5140) | 4012 (2653-5694) |
| Number of ICU beds | 8 (6-14) | 8 (6-10) | 10 (8-16) |
| Annual volume of ICU patients | 595 (380-925) | 522 (316-764) | 676 (415-1066) |
| Annual volume of ICU patients per one ICU bed | 64 (49-84) | 64 (48-86) | 63 (50-82) |
| ICU bed occupancy | 67.4 (54.6-76.9) | 67.9 (56.5-77.0) | 66.7 (53.3-76.8) |
| Hospital with IMCU beds | 415 (70.0) | 148 (60.7) | 267 (76.5) |

DPC, Diagnosis Procedure Combination; ICU, intensive care unit; IMCU, intermediate care unit

**Supplementary Table 5.** Regional Variation in Estimated ICU, IMCU, and ICU+IMCU Costs per 100,000 Population in Japan’s 47 prefectures, 2018–2022

|  |  |  |  |  |  |  | Mean |
| --- | --- | --- | --- | --- | --- | --- | --- |
|  |  |  | Mean |  | Mean | Number of | ICU+IMCU |
|  |  | Number of | ICU cost, | Number of | IMCU cost, | ICU+IMCU | cost, |
|  |  | ICU beds, | million yen | IMCU beds, | million yen | beds, | million yen |
|  |  | per 100,000 | per 100,000 | per 100,000 | per 100,000 | per 100,000 | per 100,000 |
|  | Population | population | population | population | population | population | population |
| Subdivisions | in 2022 | in 2022 | in 2018-2022 | in 2022 | in 2018-2022 | in 2022 | in 2018-2022 |
| Hokkaido | 5260000 | 4.4 | 221.1 | 17.1 | 498.1 | 21.5 | 719.1 |
| Aomori-ken | 1254000 | 5.4 | 264.6 | 10.4 | 308.7 | 15.8 | 573.3 |
| Iwate-ken | 1222000 | 2.8 | 131.7 | 13.3 | 327.6 | 16.1 | 459.3 |
| Miyagi-ken | 2310000 | 5.5 | 253.0 | 13.4 | 414.8 | 18.9 | 667.8 |
| Akita-ken | 972000 | 3.7 | 233.7 | 9.8 | 432.1 | 13.5 | 665.7 |
| Yamagata-ken | 1077000 | 3.0 | 154.2 | 12.6 | 471.3 | 15.6 | 625.5 |
| Fukushima-ken | 1852000 | 6.3 | 226.3 | 9.0 | 299.2 | 15.3 | 525.4 |
| Ibaraki-ken | 2877000 | 5.1 | 221.6 | 13.9 | 318.7 | 19.0 | 540.3 |
| Tochigi-ken | 1941000 | 4.7 | 229.7 | 14.4 | 427.4 | 19.1 | 657.1 |
| Gumma-ken | 1948000 | 2.6 | 446.7 | 13.1 | 400.4 | 15.7 | 847.1 |
| Saitama-ken | 7343000 | 4.2 | 212.2 | 13.6 | 381.5 | 17.8 | 593.7 |
| Chiba-ken | 6284000 | 6.2 | 285.6 | 12.4 | 363.6 | 18.6 | 649.1 |
| Tokyo-to | 14006000 | 7.7 | 395.5 | 15.6 | 516.4 | 23.3 | 911.9 |
| Kanagawa-ken | 9228000 | 5.4 | 264.0 | 14.1 | 433.4 | 19.5 | 697.4 |
| Niigata-ken | 2223000 | 1.4 | 73.5 | 10.9 | 406.7 | 12.3 | 480.2 |
| Toyama-ken | 1044000 | 3.6 | 162.3 | 12.8 | 323.2 | 16.4 | 485.5 |
| Ishikawa-ken | 1138000 | 4.6 | 236.3 | 16.8 | 423.7 | 21.4 | 660.0 |
| Fukui-ken | 768000 | 5.3 | 245.2 | 13.0 | 346.4 | 18.3 | 591.6 |
| Yamanashi-ken | 813000 | 2.7 | 120.9 | 7.0 | 251.3 | 9.7 | 372.2 |
| Nagano-ken | 2060000 | 5.3 | 236.8 | 19.7 | 618.4 | 25.0 | 855.2 |
| Gifu-ken | 1993000 | 3.7 | 169.9 | 12.1 | 366.0 | 15.8 | 536.0 |
| Shizuoka-ken | 3652000 | 4.1 | 199.1 | 16.0 | 508.4 | 20.1 | 707.5 |
| Aichi-ken | 7557000 | 5.2 | 243.3 | 12.6 | 451.5 | 17.8 | 694.8 |
| Mie-ken | 1784000 | 3.3 | 141.2 | 15.2 | 380.1 | 18.5 | 521.3 |
| Shiga-ken | 1417000 | 4.4 | 200.9 | 13.3 | 425.2 | 17.7 | 626.1 |
| Kyoto-fu | 2591000 | 6.1 | 316.4 | 16.7 | 485.4 | 22.8 | 801.8 |
| Osaka-fu | 8843000 | 7.3 | 394.0 | 15.5 | 502.8 | 22.8 | 896.9 |
| Hyogo-ken | 5488000 | 7.1 | 306.8 | 13.5 | 408.8 | 20.6 | 715.6 |
| Nara-ken | 1335000 | 6.1 | 347.1 | 17.4 | 480.4 | 23.5 | 827.5 |
| Wakayama-ken | 935000 | 6.3 | 247.4 | 12.8 | 395.8 | 19.1 | 643.2 |
| Tottori-ken | 558000 | 4.7 | 257.7 | 18.8 | 482.2 | 23.5 | 739.9 |
| Shimane-ken | 675000 | 6.1 | 349.7 | 12.5 | 381.5 | 18.6 | 731.2 |
| Okayama-ken | 1899000 | 12.1 | 552.6 | 16.7 | 405.5 | 28.8 | 958.1 |
| Hiroshima-ken | 2813000 | 3.6 | 217.9 | 11.0 | 375.6 | 14.6 | 593.5 |
| Yamaguchi-ken | 1354000 | 6.9 | 289.6 | 14.8 | 397.5 | 21.7 | 687.1 |
| Tokushima-ken | 731000 | 4.7 | 232.2 | 17.2 | 535.1 | 21.9 | 767.3 |
| Kagawa-ken | 959000 | 6.9 | 291.2 | 19.9 | 520.1 | 26.8 | 811.4 |
| Ehime-ken | 1349000 | 5.8 | 234.3 | 13.8 | 406.3 | 19.6 | 640.6 |
| Kochi-ken | 699000 | 7.7 | 503.6 | 27.3 | 735.6 | 35.0 | 1239.2 |
| Fukuoka-ken | 5137000 | 7.5 | 319.2 | 19.7 | 582.6 | 27.2 | 901.8 |
| Saga-ken | 813000 | 4.9 | 218.8 | 17.9 | 544.7 | 22.8 | 763.5 |
| Nagasaki-ken | 1327000 | 5.0 | 228.8 | 14.0 | 437.2 | 19.0 | 666.0 |
| Kumamoto-ken | 1750000 | 5.1 | 300.0 | 18.4 | 632.7 | 23.5 | 932.8 |
| Oita-ken | 1135000 | 3.9 | 187.6 | 10.7 | 393.8 | 14.6 | 581.5 |
| Miyazaki-ken | 1078000 | 4.6 | 220.5 | 9.1 | 281.7 | 13.7 | 502.2 |
| Kagoshima-ken | 1603000 | 6.1 | 301.4 | 13.8 | 456.4 | 19.9 | 757.8 |
| Okinawa-ken | 1460000 | 9.5 | 469.7 | 17.7 | 534.1 | 27.2 | 1003.8 |

ICU, intensive care unit; IMCU, intermediate care unit

**Supplementary Figure 1.** Correlation between Regional ICU and IMCU Cost Estimates and Corresponding Bed Densities in Japan

ICU, intensive care unit; IMCU, intermediate care unit.

Scatter plots showing the relationships between mean annual ICU, IMCU, and combined (ICU + IMCU) costs per 100,000 population and corresponding bed densities across Japan’s 47 prefectures. Each point represents one prefecture. Linear regression lines with 95% confidence intervals are shown. ICU and IMCU costs were strongly correlated with their respective bed densities (r = 0.88 and r = 0.90; both p < 0.001), and the correlation remained robust when the two units were combined (r = 0.92; p < 0.001).
